# 胸外科术后重症患者下肢深静脉血栓形成及其凝血状态分析

**DOI:** 10.3779/j.issn.1009-3419.2018.11.12

**Published:** 2018-11-20

**Authors:** 莹 黄, 春梅 王, 毅 张, 雅婵 宁, 立兵 隗, 礼坡 宋, 修益 支, 丹 鄢, 训明 吉

**Affiliations:** 1 100053 北京，首都医科大学宣武医院血管外科监护室 Department of Vascular Surgery Intensive Care Unit, Xuanwu Hospital, Capital Medical University, Beijing 100053, China; 2 100053 北京，首都医科大学宣武医院胸外科 Department of Thoracic Surgery, Xuanwu Hospital, Capital Medical University, Beijing 100053, China; 3 100053 北京，首都医科大学宣武医院介入放射科 Department of Interventional Radiography, Xuanwu Hospital, Capital Medical University, Beijing 100053, China; 4 100038 北京，北京世纪坛医院药剂科 Department of Pharmacy, Beijing Shijitan Hospital, Beijing 100038, China

**Keywords:** 胸外科手术, 术后, 深静脉血栓, 凝血, 血栓弹力图, Thoracic surgery, Postoperation, Deep venous thrombosis, Coagulation analysis, Thrombelastogram

## Abstract

**背景与目的:**

分析胸外科术后重症患者住重症加强护理病房（intensive care unit, ICU）期间的下肢深静脉血栓（deep venous thrombosis, DVT）发生率与凝血指标。

**方法:**

回顾性分析2016年7月-2018年6月接受物理预防血栓的胸外科术后重症患者，将床旁下肢静脉超声、血常规、凝血四项、血栓弹力图等结果完善的病例纳入研究，对发生血栓人群的一般信息、病种、手术方式、凝血指标等进行分析。

**结果:**

共纳入患者50例，年龄22岁-80岁，男34例，女16例。共有11例患者出现小腿深静脉血栓（22.0%），男性发生术后血栓的比例为29.4%（10/34），女性为6.3%（1/16）。恶性肿瘤的DVT发生率为23.5%（8/34），非恶性肿瘤的发生率为18.6%（3/16）。比较发生血栓和未发生血栓组的血小板计数、凝血四项、血栓弹力图等，除活化部分凝血活酶时间血栓组较无血栓组显著延长外，余无统计学差异。

**结论:**

即便在充分物理预防的基础上，胸科术后重症患者下肢深静脉血栓仍为高发，且男性、恶性肿瘤患者多见；术后超声筛查有助于早期发现DVT。

## Abstract (en)

**Background and objective:**

The aim of this study was to analyze the incidence of lower limb deep venous thrombosis (DVT) and the corresponding coagulation status in severe patients after thoracic surgery.

**Methods:**

Severe patients after thoracic surgery who received mechanical prophylaxis of lower limb DVT between July 2016 and June 2018 were analysed retrospectively. Their general information, disease species, surgical treatment, and coagulation index were reviewed.

**Results:**

Fifty patients were finally included. There were 34 male and 16 female, aging from 22 to 80 years. The incidence of DVT was 22.0%, all of them were isolated calf DVT. The incidence was 29.4% in male patients, while 6.3% in female; 23.5% in malignant diseases and 18.6% in benign. All coagulation index presented no statistical difference between patients with and without DVT, except activated partial thromboplastin time (APTT).

**Conclusion:**

Even on the basis of adequate mechanical prophylaxis, lower limb DVT is common in severe patients after thoracic surgery. Meanwhile, male patients and malignant diseases are more suscepted.

静脉血栓栓塞症（venous thromboembolism, VTE）是外科手术后的常见并发症，VTE包括下肢深静脉血栓（deep venous thrombosis, DVT）和肺动脉栓塞（pulmonary embolism, PE），下肢DVT是PE最常见的来源^[1]^。由于术后VTE多无症状，起病隐匿，常常在临床上被忽视。随着诊断技术的发展，研究发现胸外科术后VTE作为最严重的并发症之一，其发生率并不容忽视^[2]^。

本文对我院接受胸外科手术并且术后入住重症加强护理病房（intensive care unit, ICU）的患者的DVT发病情况及凝血状态进行了分析。现将结果报道如下。

## 1 资料与方法

### 1.1 一般资料

回顾性分析2016年7月-2018年6月期间因胸外科手术入外科ICU并停留时间≥72 h的患者的资料，所有患者均接受了物理方法预防DVT（抗血栓压力带或间歇性压力驱动装置），均无下肢深静脉置管，对完善下肢静脉超声和凝血功能、血栓弹力图的患者纳入研究。数据收集包括性别、年龄、体质量指数、诊断、手术名称、血常规、凝血功能、血栓弹力图等。

### 1.2 方法

术后48 h内完成血常规、凝血四项、D-二聚体及血栓弹力图检查，采血前患者均未接受抗血小板或抗凝治疗。入住ICU期间，每3天对患者进行下肢深静脉超声筛查，以静脉管腔不能压闭或仅能部分压闭、血流不能完全充盈、管腔内见异常回声为DVT的诊断标准。

### 1.3 统计学方法

采用SPSS 17.0分析数据，计数资料采用率（%）表示，计量资料采用均数±标准差（Mean±SD）表示，组间比较采用*t*检验，以*P* < 0.05为差异有统计学意义。

## 2 结果

### 2.1 一般资料

本研究共纳入患者50例，其中男性34例，女性16例（男女比例2.13:1），平均年龄（60.29±13.72）岁（22岁-80岁）。肺恶性肿瘤22例，食管恶性肿瘤12例，重症肌无力合并胸腺瘤/胸腺增生11例，肺良性病变5例。手术方式包括：胸腔镜下肺叶切除18例，胸腔镜辅助小切口左全肺切除2例，胸膜活检固定术3例，胸腔镜下肺楔形切除术4例，开放食管手术8例，胸腹腔镜辅助食管切除4例，正中开胸扩大胸腺切除2例，胸腔镜下扩大胸腺切除/纵隔肿物切除9例。

### 2.2 术后下肢DVT发生率

共有11例患者出现下肢DVT（22%），累及15条肢体（左下肢5例，右下肢2例，双下肢4例），均为小腿深静脉血栓，其中累及肌间静脉者10例，累及胫后静脉/腓静脉者2例，发现下肢DVT时间为5.2 d（1 d-14 d）。

11例患者中，男性10例，女性1例，男女比例10:1，男性发生术后DVT的比例为29.4%（10/34），女性为6.3%（1/16）。肺恶性肿瘤者6例（6/22, 27.3%），食道恶性肿瘤2例（2/12, 16.7%），重症肌无力3例（3/12, 25.0%）。恶性肿瘤的DVT发生率为23.5%（8/34），非恶性肿瘤的发生率为18.6%（3/16）。

### 2.3 术后凝血功能与下肢DVT的发生

比较发生血栓和未发生血栓组的血小板计数、国际标准化比值、凝血酶原时间、活化部分凝血活酶时间、凝血酶时间、纤维蛋白原、D-二聚体、血栓弹力图的R值、K值、α角和MA值，除活化部分凝血活酶时间发生血栓组较未发生组显著延长外[(42.3±4.27) s *vs* (38.3±4.11) s, *P*=0.022]，余均无统计学差异（表 1）。

**1:** 发生下肢DVT和未发生下肢DVT患者凝血指标比较 Comparison of coagulation indices between lower extremity deep venous thrombosis (DVT) and non-DVT patients

		DVT patients	Non-DVT patients	*P*
Platelet count (×10^9^/L)		206.00±106.83	217.00±83.30	0.733
Four items of coagulation	International normalized ratio (INR)	1.15±0.05	1.11±0.09	0.227
	Activated partial thromboplastin time (s)	42.3±4.27	38.3±4.11	0.022
	Thrombin time (s)	14.8±0.86	15.4±4.23	0.684
	Fibrinogen (g/L)	4.56±1.48	4.89±1.30	0.558
D-dimer (*μ*g/mL)		3.26±2.73	2.64±3.19	0.683
Thrombus elasto graph	R (s)	5.61±0.60	6.03±2.12	0.601
	K (min)	1.46±0.31	1.43±0.54	0.908
	α angle (deg)	69.53±4.12	69.90±6.29	0.882
	MA (mm)	62.44±6.13	65.36±6.28	0.273

## 3 讨论

VTE是外科术后常见并发症之一，虽然近年来越来越受到重视，但在胸外科术后患者VTE的防治方面，还缺乏权威的指南或共识，临床实际上对胸外科术后VTE的发生率及防治现状亦不容乐观^[3]^。

本组患者的下肢DVT发生率为22.0%，略高于国内其他文献报道^[4]^，其中肺恶性肿瘤术后下肢DVT发生率为27.3%，这与本组患者均入住ICU时间较长、病情相对较重、卧床时间延长有关。但本组所有患者均接受了物理方法预防下肢DVT，发生率仍如此之高，确实值得重视并有必要探讨进一步采用药物预防DVT的价值。发生下肢DVT的11例患者均为小腿深静脉血栓，此类血栓临床上往往没有任何症状，但若不加干预，相当一部分患者会进展至大腿血栓甚至发生肺栓塞，而抗凝治疗能够阻止其进展^[5, 6]^。

本研究中恶性肿瘤的DVT发生率高于非恶性肿瘤的DVT发生率（23.5% *vs* 18.6%），且男性在术后发生DVT的患者中占绝大多数（10/11），男性发生术后DVT的比例为29.4%，女性为6.3%，这与余开颜、任康奇等^[7, 8]^的研究有相似之处，但后者亦未能发现性别在统计学上的差异。

本研究对比了两组患者的不同方法测量的凝血状态，除了活化部分凝血活酶时间发生血栓组较未发生血栓组明显延长外，余均无统计学差异。但值得关注的是，凝血功能检测中，除了D-二聚体外，其余指标在发生血栓组均较未发生血栓组倾向于低凝，而在血栓弹力图检测中，反映凝血因子活性的R值，发生血栓组较未发生血栓组倾向于高凝。分析其原因，可能是由于凝血功能检测仅对血浆进行分析，缺少了血小板的相互作用，而血栓弹力图对全血进行分析，后者更能够反映患者体内全面、真实的凝血状态^[9, 10]^。当然，该假设仍须更大样本量研究验证，其机制亦须进一步深入研究。D-二聚体阴性虽然可以除外血栓形成，但由于术后大多数患者的D-二聚体也会升高^[11]^，难以将D-二聚体作为术后筛查血栓形成的指标，而床旁静脉超声检测是一种无创、便捷、经济的筛查手段，对于早期发现DVT并及时干预有着重要的积极意义。

本研究作为回顾性研究有其固有的局限性，样本量偏少使结果可能产生一些偏倚。本文通过总结胸科术后重症患者下肢DVT的发生率，揭示了下肢DVT在胸科术后危重患者高发的现状，提示了胸科术后患者进行血栓筛查的必要性；通过对不同方法测得的凝血指标进行分析，认为血栓弹力图可能更有助于判断体内真实的凝血因子活性，但仍需更大样本的研究和对其机制进行深入研究。
